# Draft Genome Sequence and *De Novo* Assembly of a Fusarium oxysporum f. sp. *lycopersici* Isolate Collected from the Andean Region in Colombia

**DOI:** 10.1128/mra.00980-21

**Published:** 2022-01-06

**Authors:** Pei-Ling Yu, James C. Fulton, Sandra L. Carmona, Diana Burbano-David, Luz Stella Barrero, Jose C. Huguet-Tapia, Jeremy T. Brawner, Mauricio Soto-Suarez

**Affiliations:** a Department of Plant Pathology, University of Florida, Gainesville, Florida, USA; b Corporación colombiana de investigación agropecuaria (AGROSAVIA), Mosquera, Cundinamarca, Colombia; Vanderbilt University

## Abstract

We report a draft genome assembly of the causal agent of tomato vascular wilt, Fusarium oxysporum f. sp. *lycopersici* isolate 59, obtained from the Andean region in Colombia.

## ANNOUNCEMENT

Fusarium oxysporum f. sp. *lycopersici* is a soilborne fungus belonging to the F. oxysporum species complex (FOSC). F. oxysporum f. sp. *lycopersici* causes fusarium wilt in tomato (Solanum lycopersicum), which often leads to significant yield losses (1, 2). F. oxysporum f. sp. *lycopersici* isolate 59 was isolated from root and stem tissue from a wilted tomato plant grown in the Andean region of Colombia (3). Isolate 59 was classified as F. oxysporum f. sp. *lycopersici* race 2, using PCR markers for phylogenetic analysis (3).

For whole-genome sequencing, fungal hyphae from a 6-day-old culture (Czapek-Dox medium) were collected and lyophilized overnight. High-molecular-weight (HMW) DNA was extracted using a modified phenol-chloroform/isoamyl alcohol method (4). For Nanopore sequencing, a library was prepared using the ligation sequencing kit (SQK-LSK109) according to the manufacturer’s instructions (Oxford Nanopore Technologies, Oxford, UK) using 1 μg HMW DNA. The long-fragment buffer (LFB) supplied in the kit was used to enrich long DNA fragments of >3 kb. An R9.4.1 flow cell (Oxford Nanopore Technologies) was loaded and run for 24 h. Base calling was performed using Guppy version 4.0.21 within MinKNOW (Oxford Nanopore Technologies). Illumina sequencing was performed using a fungal sample collected as previously described. Total DNA was isolated using the cetyltrimethylammonium bromide (CTAB) protocol (5). DNA (350 ng/μL) was used for library preparation with the Nextera DNA Flex library preparation kit in dual index format (Illumina, Inc., San Diego, CA, USA) according to the manufacturer’s instructions. The library was sequenced in paired-end format on the Illumina HiSeq 4000 sequencing system (Macrogen, South Korea).

The quality of the Nanopore and Illumina reads was assessed via NanoPlot version 1.30.1 (6) and FastQC version 11.7 (http://www.bioinformatics.babraham.ac.uk/projects/fastqc/), respectively. A total of 1,742,231 raw reads were generated from the Nanopore sequencing. Approximately 16 million 151-bp paired-end reads were obtained from the Illumina sequencing. The resulting long reads were first processed using Porechop version 0.2.4 to divide chimeric sequences (https://github.com/rrwick/Porechop) (7); then, the reads were filtered by length and quality using Filtlong version 0.2.0 (https://github.com/rrwick/Filtlong). The *N*_50_ length of the Nanopore reads was 9.569 kbp. A total of 885,847 filtered reads were assembled using the *de novo* long-read assembler Shasta version 0.1.0 (8). The sequenced short reads were processed by first removing residual adapters and poor quality reads using Trim Galore version 0.6.5 (https://www.bioinformatics.babraham.ac.uk/projects/trim_galore/). Reads shorter than 100 bp were filtered using the FASTX-Toolkit version 0.0.14 (fastx_trimmer; http://hannonlab.cshl.edu/fastx_toolkit). The *de novo* assembly Nanopore and Illumina reads were polished using Racon version 1.4.13 (9) and Pilon (10), respectively. Whole-genome assembly was carried out using a hybrid *de novo* assembly approach, incorporating Nanopore long reads and Illumina short reads.

**FIG 1:**
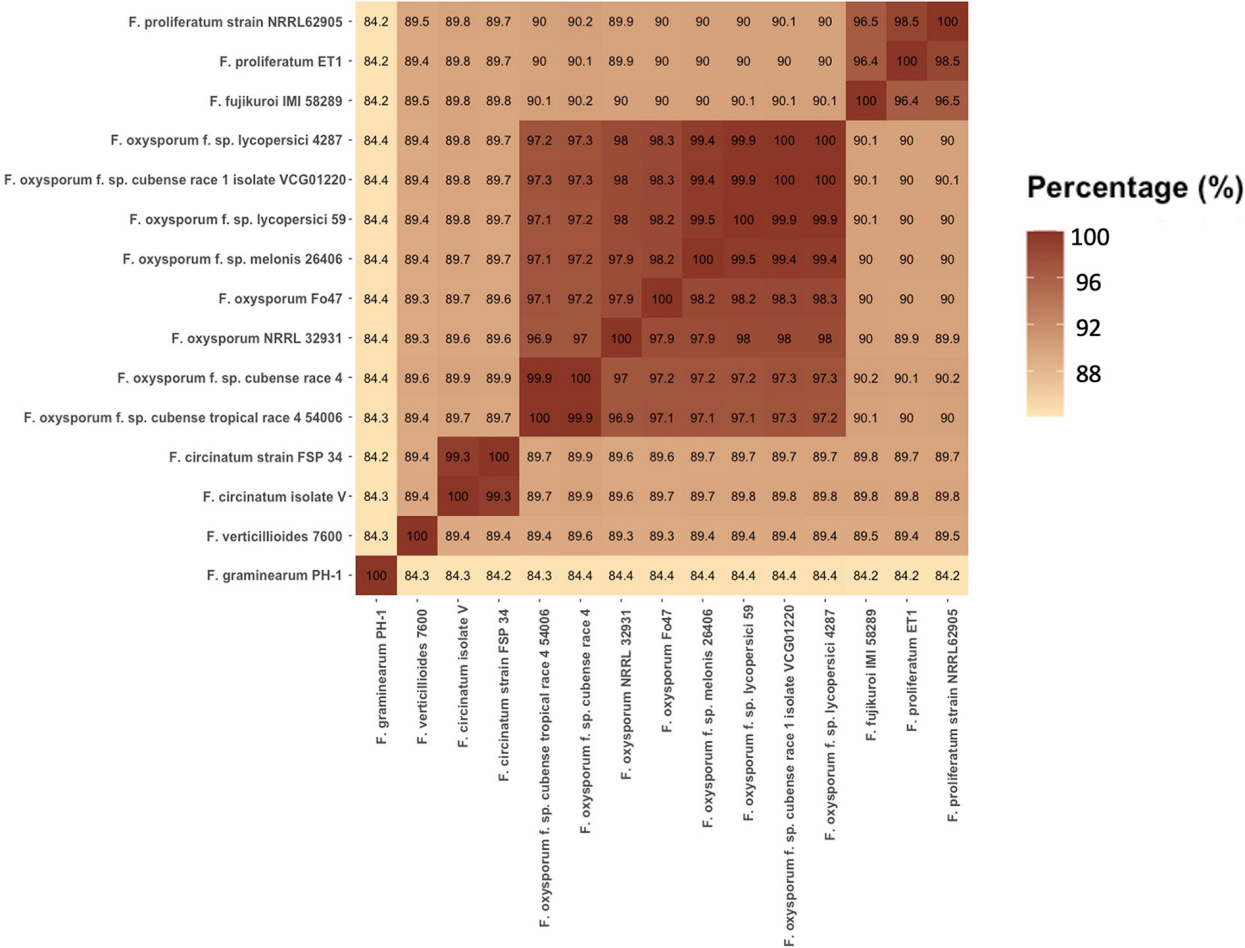
Heatmap table of the average nucleotide identity (ANI) values generated from a pairwise comparison of 15 Fusarium isolates. An ANI score greater than 95% between two genomes indicates that they are the same species. The genomes of the Fusarium isolates were downloaded from NCBI: F. oxysporum f. sp. *cubense* race 4 (GenBank accession number GCA_000350365.1), F. circinatum strain FSP 34 (GCA_000497325.3), F. oxysporum f. sp. *melonis* 26406 (GCA_002318975.1), F. oxysporum Fo47 (GCA_013085055.1), F. circinatum isolate V (GCA_013168815.1), F. oxysporum f. sp. *cubense* race 1 isolate VCG01220 (GCA_016802225.1), F. proliferatum strain NRRL62905 (GCA_900029915.1), F. verticillioides 7600 (GCF_000149555.1), F. oxysporum f. sp. *lycopersici* 4287 (GCA_003315725.1), F. graminearum PH-1 (GCF_000240135.3), F. oxysporum f. sp. *cubense* tropical race 4 strain 54006 (GCF_000260195.1), F. oxysporum NRRL 32931 (GCF_000271745.1), F. proliferatum ET1 (GCF_900067095.1), *F. fujikuroi* IMI 58289 (GCF_900079805.1).

A summary of assembly statistics was generated using BBMap version 38.90 (11), and the assembly completeness was evaluated using the Benchmarking Universal Single-Copy Orthologs (BUSCO) version 4.0.6 software (12) (Table 1). PYANI version 0.2.10 was used to calculate the average nucleotide identity (ANI) and relatedness measures of whole-genome comparisons among Fusarium species (13) (Fig. 1). The draft assembly (combining long reads and Illumina short reads) has a total size of 54.2 Mb and a coverage of approximately 75.5×. The completeness of the assembly was calculated using BUSCO with the Hypocreales_odb10 lineage gene data set; the analysis showed that 4,441 out of 4,494 BUSCO markers were found, and only a few duplicated or missing BUSCO orthologs were identified (Table 1).

**TABLE 1:** Comparison of assembly statistics of Fusarium oxysporum f. sp. *lycopersici* isolates

Characteristic	Data for F. oxysporum f. sp. *lycopersici* strain:		
	59	4287	4287
Accession no. (database)	PRJNA756266 (BioProject)	GCF_000149955.1 (GenBank assembly)	GCA_003315725.1 (GenBank assembly)
Sequencing method	Oxford Nanopore + Illumina	Sanger	PacBio + Illumina
Total length (Gbp)	5.36	6.1	5.39
No. of contigs	361	1,362	504
Coverage (×)	75.5	6.5	76
Assembly size (Mb)	54.2	59.9	53.9
Longest contig (bp)	6,457,141		5,700,000
% GC	47.67	48.4	47.7
Contig *N*_50_ (bp)	3,035,620	95,416	1,338,693
Contig *L*_50_	7	184	11
Complete BUSCOs (%)	99.60	97.70	99.90
Total no. of BUSCOs	4,494	4,494	2,294
No. of duplicate BUSCOs	37	40	34
No. of fragmented BUSCOs	0	24	1
No. of missing BUSCOs	7	78	7
Reference	This study	15	14

The results of this study will contribute to building a more robust phylogenetic framework that will guide inquiries concerning the evolution of important traits in the FOSC group.

### Data availability.

The described genome assembly is available in GenBank under BioProject accession number PRJNA756266. The Illumina and Oxford Nanopore reads are deposited at the Sequence Read Archive (SAR) under accession numbers SRX11976571 and SRX11976570, respectively. F. oxysporum f. sp. *lycopersici* strain 59 was registered in the National Collections Registry (RNC129) and was collected under AGROSAVIA permit framework number 1466 from 2014, updated by resolution 04039 on 19 July 2018.
